# Navigational affordances emerge stably with brief stimulus exposure despite changes in scene content

**DOI:** 10.1167/jov.26.10.17

**Published:** 2026-10-14

**Authors:** Edward H. Silson

**Affiliations:** 1 Department of Psychology, School of Philosophy, Psychology & Language Sciences, University of Edinburgh, Edinburgh, UK

**Keywords:** navigational affordances, brief exposure, invariant, stable representations, complex scenes

## Abstract

Everyday life requires successful navigation often through complex and dynamic environments. The ability to identify efficient and safe routes of egress through scenes relies on perception of the navigational affordances of scenes. Recent behavioral work identified that navigational affordances of indoor urban environments can emerge with as little as 33 to 66 ms of stimulus presentation but only become stable with 132 ms and beyond. Here, we extend this to investigate the temporal emergence of navigational affordances using a more heterogeneous stimulus set that includes both indoor urban and outdoor environments (both person-made and natural). We observed an almost identical pattern of results to prior work: navigational affordances were emergent rapidly (∼66 ms) and stabilized with as little as 132 ms of stimulus exposure. This pattern was also evident when considering the outdoor scenes in isolation. Taken together, these data suggest that the human visual system is capable of forming stable navigationally relevant representations for maximal behavioral relevance rapidly.

## Introduction

A seemingly ubiquitous aspect of human life is the need to successfully navigate through the local environment, identifying safe and efficient paths for navigation whilst also avoiding obstacles (e.g., other people or things). To achieve this, the human visual system is required to efficiently interpret and process a dynamically shifting and complex visual environment and to make such computations available to inform behavior.

Prior work has highlighted how the human visual system appears ideally tuned to extract navigationally relevant visual features efficiently and/or automatically (Bartnik et al., 2025; Bonner & Epstein, 2017; Dwivedi, Cichy, & Roig, 2020; Greene & Oliva, 2009; Potter, 1975; Thorpe, Fize, & Marlot, 1996; Zamboni et al., 2026). For example, the navigability of scenes (whether a scene is navigable or not) can be discerned accurately (75% correct) with a stimulus duration of only ∼34 ms (Greene & Oliva, 2009). Likewise, basic-level scene categorization, such as whether a scene is a lake or a beach can be performed accurately within 50-100 ms (Greene & Oliva, 2009). These behavioral results demonstrate how the visual system is capable of complex visual scene analysis to inform scene understanding in the briefest of glances.

Within the broad umbrella term of navigation, navigational affordances describe the ability to perceive and efficient routes of egress within the local environment. The automaticity, computational mechanisms, spatiotemporal dynamics and developmental processes underlying navigational affordances have been a source of intense investigation over the last decade or so (and likely longer) (Bartnik et al., 2025; Bonner & Epstein, 2017; Bonner & Epstein, 2018; Dwivedi et al., 2020; Harel, Nador, Bonner, & Epstein, 2022; Kamps, Chen, Kanwisher, & Saxe, 2025; Zamboni et al., 2026). Functional magnetic resonance imaging (fMRI) work of lasting importance (Bonner & Epstein, 2017) identified the scene-selective occipital place area (OPA), located on the lateral surface of occipitotemporal cortex, as the most likely locus of automatic navigational affordance coding. More recent fMRI work (Zamboni et al., 2026) replicated the finding of significant navigational affordance coding in OPA but suggested that early dorsal regions of visual cortex that fall posterior to the OPA were more likely candidates for this critical cortical locus.

In the temporal domain, electroencephalography research suggests that neural representations of navigational affordances of highly simplified artificial environments can emerge as early as ∼134 ms post stimulus onset (Harel et al., 2022). This time window aligns well with recent magnetoencephalography data that highlights a similar time-frame (∼110 ms post stimulus onset) for the emergence of navigational affordances of urban indoor environments (Zamboni et al., 2026). That said, computational work suggests a temporal order of feature extraction, such that the spatial structure of a scene (two and three dimensional) is processed first, followed by semantic content and then navigational affordances (Dwivedi et al., 2020). Taken together, the neuroimaging data suggests that neural representations of navigational affordances are likely computed within early dorsal regions of visual cortex, perhaps extending into the OPA (Bonner & Epstein, 2017; Zamboni et al., 2026) and measurable largely within 100-200 ms post stimulus onset (Dwivedi et al., 2020; Harel et al., 2022; Zamboni et al., 2026).

But does acting on navigational affordances operate on a similar time-scale? Prior work by our group demonstrated that representations of navigational affordances can emerge behaviorally with very brief stimulus presentations (33-66 ms) and that these representations stabilize with as little as 132 ms of stimulus presentation (Zamboni et al., 2026). This suggests that an approximate minimum duration of ∼132 ms is required to form stable representations of navigational affordance that could be maximally beneficial to navigational behavior—but how generalizable is this? One obvious and justifiable critique of this prior work was the use of a relatively small and homogenous set of stimuli (Bonner & Epstein, 2017; Zamboni et al., 2026). Each of the 50 scenes used previously were indoor urban environments with clearly identifiable (though variable) routes of egress. Thus the current study sought to test whether the same pattern of early emergence yet stable navigational affordance representations with 132 ms of exposure would occur when the stimulus set was more heterogeneous and including both urban indoor environments and outdoor environments that were either natural or person-made.

To preview, we found a striking similarity in the pattern of navigational affordance representations across experiments. First, significant acting on navigational affordances emerged with as little as 66ms of stimulus exposure and stable navigational affordance representations emerged at 132 ms and beyond in the more heterogeneous stimulus set with remarkable consistency to prior work. These patterns remained even when considering outdoor scenes in isolation. Taken together, these behavioral data suggest that representations of navigational affordances emerge rapidly (∼33–66 ms) and stabilize with brief stimulus exposures (132 ms) that are largely invariant to scene content.

## Methods

Participants: 144 participants split across two experiments completed the navigational affordance task. Experiment 1: 72 participants (52 female) with normal or corrected-to-normal vision and a mean age of 28.51 (±4.10) completed the navigational affordance behavioral experiment as reported in (Zamboni et al., 2026), which we here refer to as Experiment 1. All participants completed the task in one condition only (six condition, *n* = 12 per condition). All participants provided informed consent and were compensated monetarily for their participation. Experiment 2: An independent group of 72 participants (61 female) with normal or corrected-to-normal vision and a mean age of 27.21 (±5.46) completed the current navigational affordance behavioral experiment, which we here refer to as Experiment 2. Ethical approval for both experiments was provided by the School of Philosophy, Psychology and Language Sciences ethics committee at the University of Edinburgh (Ethical application number 42-2425/7).

### Visual stimuli and tasks

Experiment 1: Participants were presented with the same 50 scene images as used previously (Bonner & Epstein, 2017; Bonner & Epstein, 2018; Dwivedi et al., 2020; Zamboni et al., 2026). Experiment 2: Participants were presented with a different set of 50 scenes that included both outdoor (person-made/natural) and indoor scenes (25 of each). These stimuli were taken from prior work (Bartnik et al., 2025). Figure 1 depicts exemplar stimuli from both experiments.

**Figure 1.**
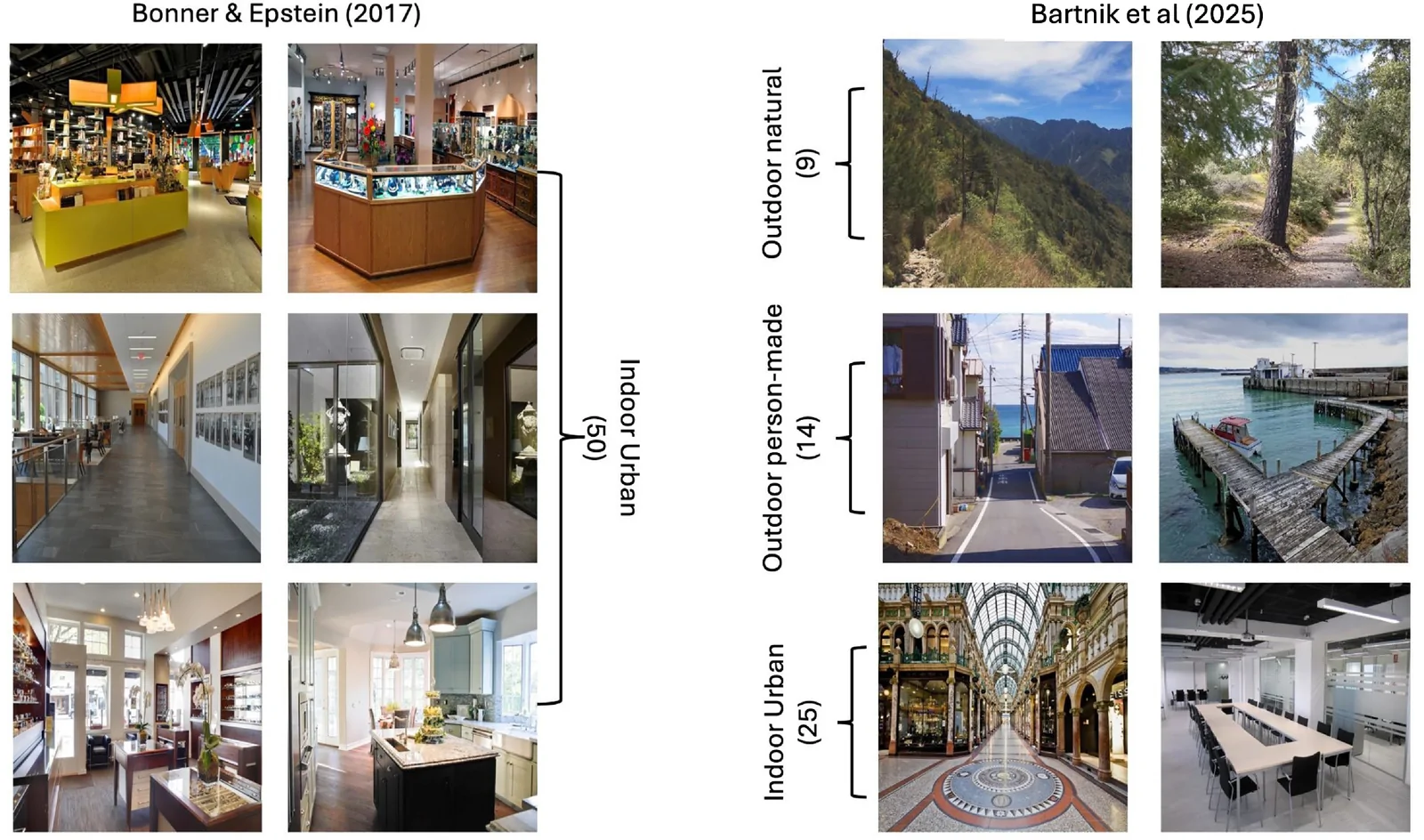
navigational affordance stimuli: (Left): Images depict six scenes (out of 50) used originally in (Bonner & Epstein, 2017) and subsequently (Dwivedi et al., 2020; Zamboni et al., 2026). These scenes depict indoor urban environments with clear yet variable routes of egress. (right): Images depict six scenes (out of 50) taken from (Bartnik et al., 2025) and used in Experiment 2. Here, scenes can be broken down into three distinct semantic categories: Outdoor natural (*n* = 9, top-right), Outdoor person-made (*n* = 14, middle-right) and indoor urban (*n* = 25, bottom-right).

The two stimulus sets differ across several dimensions. First, all 50 scenes from Bonner & Epstein (2017) depict indoor urban environments with clear and unambiguous paths for navigation that lead largely to the left, straight forwards, to the right or a combination of these main trajectories. In contrast, the stimuli from Bartnik et al (2025) depict scenes with more ambiguous navigational paths, particularly for the outdoor scenes. For example, given a beach scene, one could perceive navigational affordances in any direction (including into the water). Second, because of the more varied nature of the environments depicted in the Experiment 2 stimulus set, the types of visual feature and the spatial distribution of these features are more varied as compared to the Experiment 1 stimulus set. All 50 scenes used in Experiment 2 are shown in Figure 2.

**Figure 2.**
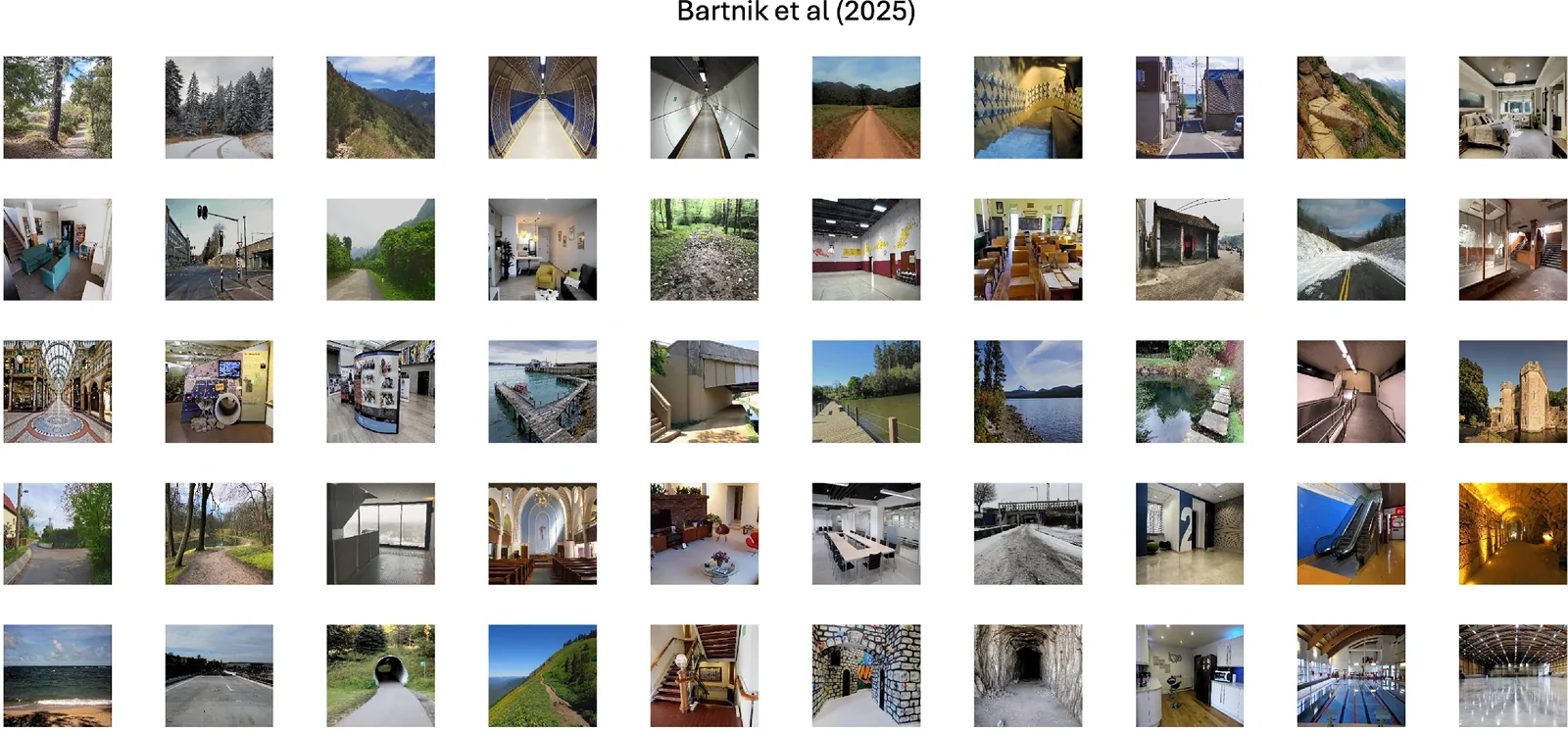
All 50 scenes used in Experiment 2, sampled from Bartnik et al (2025).

Both experiments followed the exact same procedure (Figure 3A). Following a variable inter-trial interval (1–1.5 s), a scene (17° × 13°) was presented briefly (either 33, 66, 132, 264, or 528 ms) followed by a dynamic mask (5  ×  50 ms). Post mask, the border of the image remained in red and participants were asked to draw the available routes of egress by moving the mouse across the screen. The baseline conditions of both experiments were similar apart from the lack of a dynamic mask as the scene remained visible throughout the path drawing phase. The start position of the mouse was fixed on each trial to be the center and bottom of the scene (unrestricted condition) or scene border (time-dependent conditions). Mask stimuli were created by first dividing each scene (768  ×  1024 pixels) into 64 equal sized rectangles (96  ×  128 pixels) before shuffling rectangles both within and across scenes.

**Figure 3.**
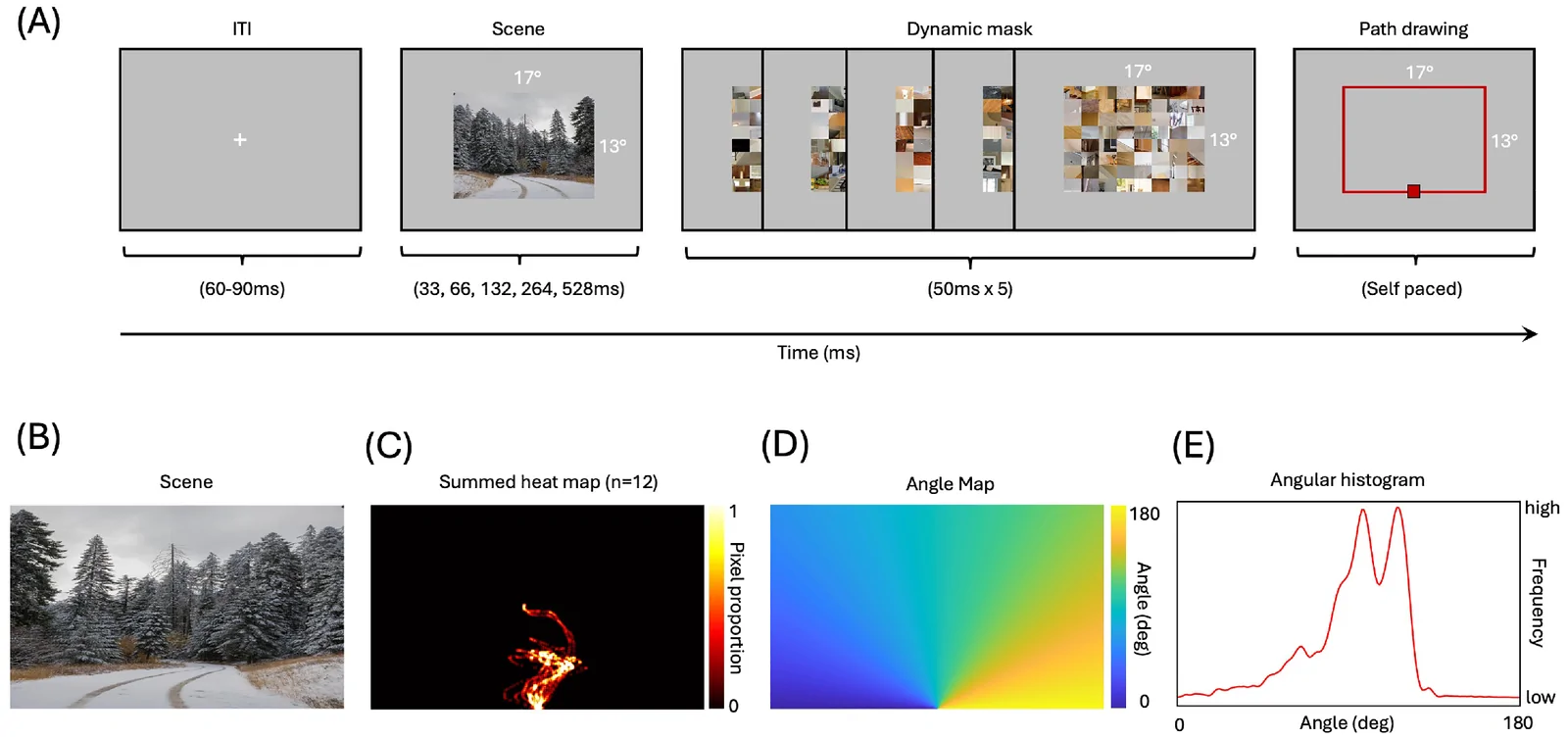
Task schematic and analysis pipeline: (A) Schematic of the time-dependent task used in Experiments 1 and 2. Following a variable inter-trial-interval, a scene (17° × 13°) was presented briefly (either 33, 66, 132, 264, or 528 ms) followed by a dynamic mask (5 × 50 ms). Post mask, the outline of the image remained in red, and participants were asked to draw the available routes of egress. The start position of the mouse was fixed on each trial to be the center and bottom of the scene outline (red square). The baseline conditions were similar but were self-paced and did not include the dynamic masking or scene-outline. (B) Example outdoor natural scene used in Experiment 2. (C) Heat map of path trajectories for this scene summed across participants (*n* = 12) from the baseline condition. (D) Angle map that divides the same stimulus space into 180 equally spaced bins from angle = 0° (directly to the left along the horizontal x) to angle = 180° directly to the right along the horizontal x). (E) Angular histogram depicts the frequency of path trajectories in (C) as a function of each angular bin in (D).

Both experiments were implemented in PsychoPy (Peirce, 2007) with visual stimuli presented via a HP EliteDesk monitor (resolution: 1920  ×  1080 pixels; refresh rate: 60 Hz; visible display size: 531.36 mm  ×  298.89 mm). We did not receive reports of dropped frames during the experiment. Post data collection we nevertheless tested for dropped frames using the in-built timeByFramesEx.py module within PsychoPy. Out of a sample of 500 frames none were detected as dropped (mean refresh rate = 16.67 ms, *SD* = 0.03 ms).

An important note about the current design is that it is a between-participant design. This choice was largely made to be complementary to prior work using the visual stimuli used here.

### Analysis procedure

Analysis procedure was identical for all conditions and both experiments (Figures 3B–E) and followed the approach outlined previously (Bonner & Epstein, 2017). Analyses were conducted in MATLAB (R 2025b). The x, y pixel locations of path trajectories for each scene were extracted for each participant within a condition. Next, trajectories were spatially blurred (imfilter) before being summed across participants for each scene (Figure 3C). Summed heat maps were converted into angular histograms by calculating the summed value in each of 180 angular bins (Figures 3D, 3E). Angular histograms were then normalized (*z* score) and smoothed (smoothn). Finally, representational dissimilarity matrices (RDMs) were created by calculating the pairwise dissimilarity in the squared Euclidean distance between the angular histograms. This was done separately for each time-dependent condition (baseline, 33 ms, 66 ms, 132 ms, 264 ms, and 528 ms). To create the null distributions for each timing condition, we first randomly shuffled the time-dependent RDMs before computing the correlation (Pearson's *r*) between the shuffled RDM and the comparison RDM (e.g., baseline RDM). This procedure was repeated 10,000 times creating a null distribution of expected correlations by chance. Note, the current analysis procedure captures the overall spatial topography of navigational affordances but does not capture temporal information.

The resulting RDMs thus represent the similarity of path-based reconstructions across participants for that given time-dependent condition. Once in RDM format, we can directly compare the similarity of reconstructed path trajectories between time-dependent condition and either the baseline condition (i.e., with no time limit) or between time-dependent conditions directly. The central logic of the condition-condition analysis is that if the overall pattern of similarity of a group of scenes (based on reproduced path trajectories across participants) is similar at one presentation duration (e.g., 132 ms) to the pattern derived from a different presentation duration (e.g., 264 ms) then the representation has stabilized.

To compare the heterogeneity in image statistics between the two stimulus sets, we computed image statistics using the gist descriptor (http://people.csail.mit.edu/torralba/code/spatialenvelope/) to capture the low-level features of each scene. For each scene, a vector composed of 512 values was created by passing each scene through a series of Gabor filters across four spatial frequencies and eight orientations. The resulting vector characterizes the image in terms of the spatial frequencies and orientations at different spatial locations within the image. We computed the pairwise Euclidean distance between vectors to produce an RDM across stimuli.

## Results

Navigational affordances emerge early across heterogeneous scenes but only stabilize with exposure of 132ms and beyond recent behavioral data extended prior work (Greene & Oliva, 2009) by demonstrating that significant representations of navigational affordances are detectable with as little as 33–66ms of stimulus exposure but that these representations only stabilize with stimulus exposures of 132ms and above (Zamboni et al., 2026).

Whilst compelling, an obvious factor limiting the generalizability of this finding was the stimulus set. The stimuli, taken from prior work (Bonner & Epstein, 2017), consisted of 50 scenes depicting homogenous urban environments with clear (yet variable) routes of egress. Given this, the goal of the current study was to test whether the same pattern of early emergence and stability in navigational affordance representations would manifest with a more heterogeneous stimulus set that included three distinct semantic categories: indoor urban (*n* = 25), outdoor natural (*n* = 9), and outdoor person-made (*n* = 14). To quantify the increased heterogeneity between stimuli in Experiment 2 compared with Experiment 1 we computed the pairwise Euclidean distance in the Gist descriptor for each scene (Oliva & Torralba, 2006). The gist descriptor characterizes the image in terms of the spatial frequencies and orientations at different spatial locations within the image. It was anticipated that the mean pairwise Euclidean distance in gist descriptor would be larger (i.e., less similar) for the stimuli in Experiment 2 over the stimuli in Experiment 1. As anticipated, the heterogeneity of image statistics between scenes were larger for Experiment 2 (mean/std pairwise GIST = 0.67/0.15) compared with Experiment 1 (mean/std pairwise GIST = 0.63 / 0.14). A two-sample Kolmogorov-Smirnov test confirmed a significant difference in the distribution of pairwise Euclidean distances (ks-value = 0.15, *p* = 6.16^−14^).

In Experiment 1, we compared the time-dependent RDMs to a baseline RDM where trials were self-paced and the scenes were visible throughout the trial. This analysis identified a statistically significant, albeit weak correlation between the baseline and 33 ms condition (*r* = 0.08). The relationship with the baseline condition increased in strength substantially thereafter (66 ms: *r* = 0.22; 132 ms: *r* = 0.55; 264 ms: *r* = 0.56; 528 ms: *r* = 0.56). Thus, although a statistically significant correlation with the baseline was observed at 33 ms it was weak compared to the other conditions. Here, we performed the same comparison (i.e., Pearson's correlation) between the baseline and time-dependent RDMs for Experiment 2 to establish evidence for the earliest emergence of navigational affordance representations with a more heterogeneous stimulus set. We also compared each time-dependent RDM with the gist descriptor RDM for the same stimuli (Figure 4).

**Figure 4.**
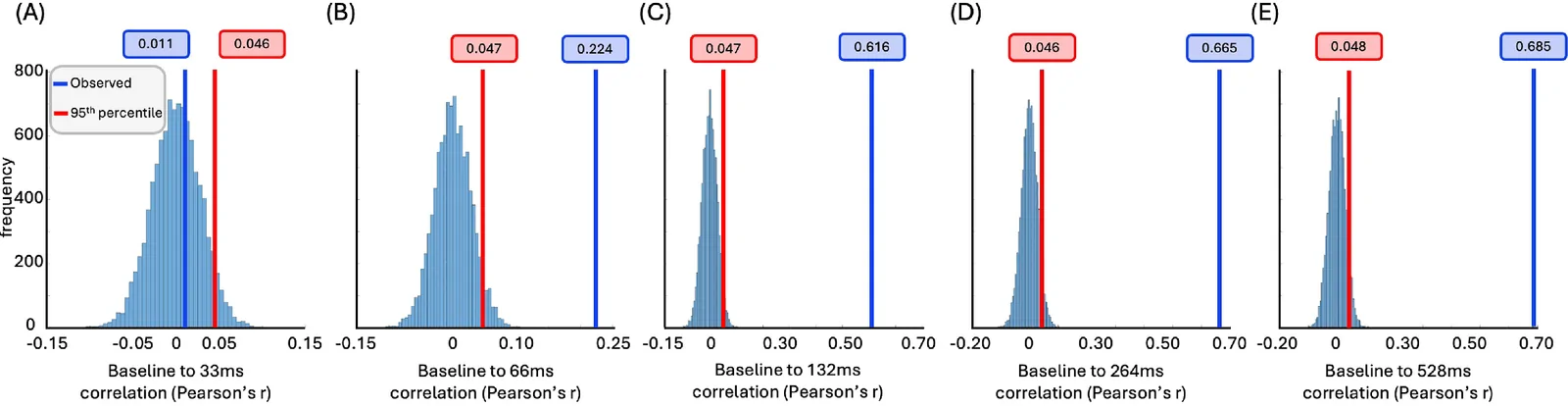
Time-dependent conditions versus baseline for Experiment 2. (A) Distribution represents the correlation (Pearson's *r*) between the baseline RDM and 10k randomly shuffled RDMs from the 33 ms condition. (B) Same as (A) but for the 66 ms condition. (C) Same as (A) but for the 132 ms condition. (D) Same as (A) but for the 264 ms condition. (E) Same as (A) but for the 528 ms condition. (All) The blue line represents the observed correlation. The red lines represent the 95th percentile of the permuted null distribution.

Across all the time-dependent conditions, we observed larger correlation coefficients with the baseline RDM over the gist RDM suggesting that the patterns observed are not likely due solely to the low-level visual features captured by the gist model. To better determine how related these similarity spaces were, we next computed the partial correlation between the baseline RDM and each time-dependent RDM while partialIng out the gist RDM. The impact on the observed correlations was very small across all time-dependent conditions. Indeed, calculating the delta between the Baseline to time-dependent correlations and when partialIng out gist did not deviate from zero strongly: 33 ms delta = −0.005; 66 ms delta = 0.0014, 132 ms delta = 0.0009; 264 ms delta = 0.0007; 528 ms delta = −0.0001.

To establish statistical significance, we shuffled each time-dependent RDM 10,000 times and computed the correlation between the shuffled RDM and the baseline RDM. This created a null distribution of the correlations with the baseline condition one could realistically expect by chance. We then compared the observed correlation with the 95th percentile of the permuted null distributions. Figures 4A–E depicts the results of this analysis for all time-dependent conditions. We observed very similar patterns to Experiment 1 in all but the quickest condition (33 ms). Unlike in Experiment 1, the observed correlation between the baseline and 33ms condition (*r* = 0.01) did not fall to the right of the 95th percentile, whereas in all other conditions it did (66 ms: *r* = 0.22; 132 ms: *r* = 0.61; 264 ms: *r* = 0.66; 528 ms: *r* = 0.68). Moreover, the correlations in both Experiment 1 and Experiment 2 show a large increase from 66 ms to 132 ms (Delta *r* Experiment 1 = 0.33; Delta *r* Experiment 2 = 0.39), which is substantially larger than the difference between any other successive conditions (next largest successive increase was 33–66 ms in Experiment 1: Delta *r* = 0.14; Experiment 2: Delta *r* = 0.21).

Taken together, the analyses described above suggest that navigational affordances can emerge with as little as 33-66 ms of stimulus exposure across scenes of different semantic types - but how stable are these representations? To test for the stability in navigational affordance representations, we compared the similarity in the path trajectories between scenes in each of the five time-dependent conditions (33, 66, 132, 264, and 528 ms) using the same approach as that applied to the path trajectories in Experiment 1 (Zamboni et al., 2026). To test the relationship between conditions we computed the pairwise dissimilarity (1-Pearson's *r*) between these time-dependent RDMs (Figure 5A). From the resulting matrix, three general patterns emerge. First, the reconstruction of navigational affordances based on (33 ms) image presentation was very dissimilar from all other conditions (mean dissimilarity of 33 ms vs. all other conditions = 0.94). Second, the representation of navigational affordances elicited at 66ms remained different from all other conditions, albeit to a lesser extent than the 33 ms condition (mean dissimilarity of 66 ms vs. all other conditions = 0.64). Third, and most crucially, the representations elicited at 132, 264, and 528 ms were highly similar to each other (mean dissimilarity between these conditions = 0.33). This pattern suggests that temporally robust navigational affordance representations of both outdoor and indoor scenes emerge with stimulus exposures of 132 ms and beyond.

**Figure 5.**
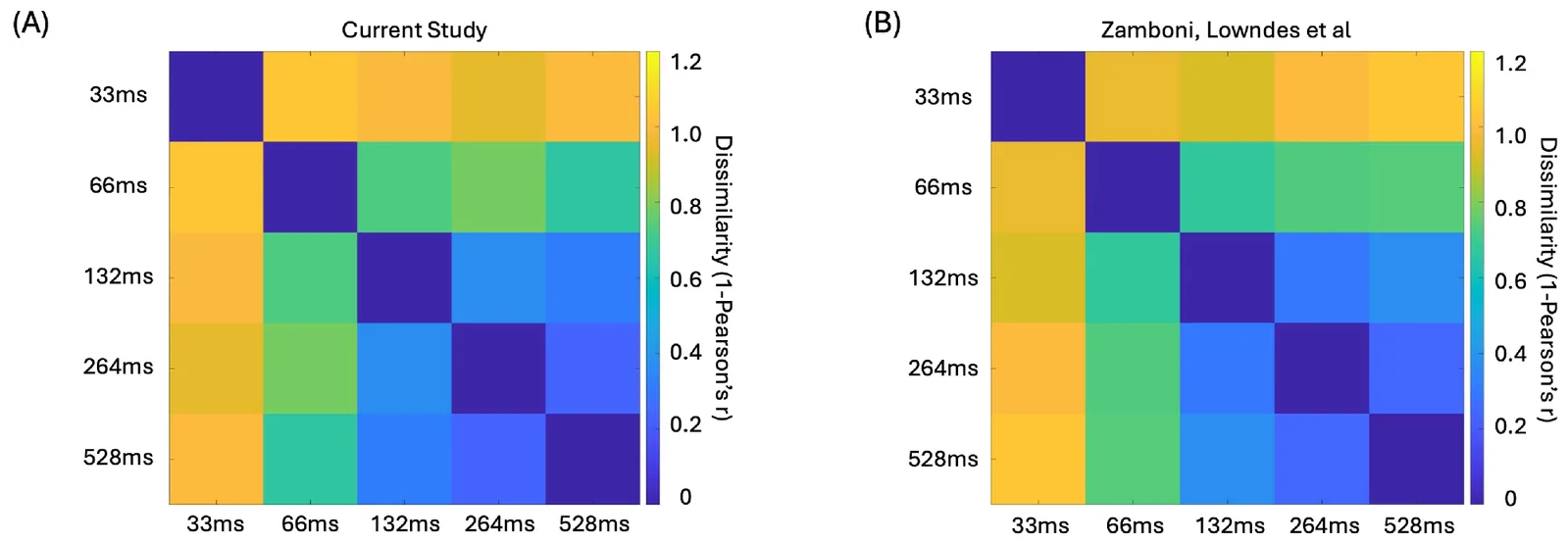
Speed of navigational affordances. (A) Time-dependent RDM-RDM matrix from the current study. Cells represent the dissimilarity (1-Pearson's r) between all pairs of time-dependent navigational affordance RDMs derived from path trajectories of both outdoor and indoor environments. (B) Same as (A) but taken from (Zamboni et al., 2026) and derived from path trajectories of indoor urban environments only.

The overall pattern between time-dependent RDMs in the current study is strikingly similar visually to the pattern between time-dependent RDMs reported previously (Zamboni et al., 2026) and shown in Figure 5B, despite substantial content differences between the stimulus sets (e.g., all indoor urban versus a mix of indoor and outdoor scenes).

To quantify this relationship, we computed the correlation (Pearson's *r*) in the pattern of dissimilarity between time-dependent conditions across both experiments by taking the lower triangle of each matrix (excluding the diagonal). These patterns were remarkably similar across experiments (*r*(9) = 0.97, *R*^2^ = 0.95, *p* = 8.65^−7^), with a linear relationship explaining ∼95% of the variance (Figure 6A). Color coding the conditions also reveals the similarity in the three major patterns that emerged within each experiment (Figure 6A). Namely, that the 33 ms condition was very dissimilar to all others (top right cluster of data points), the 66ms was more similar to the later time-points than the 33 ms condition (middle cluster of data points) yet remained separate from the 132, 264, and 528 ms conditions (bottom left cluster of data points).

**Figure 6.**
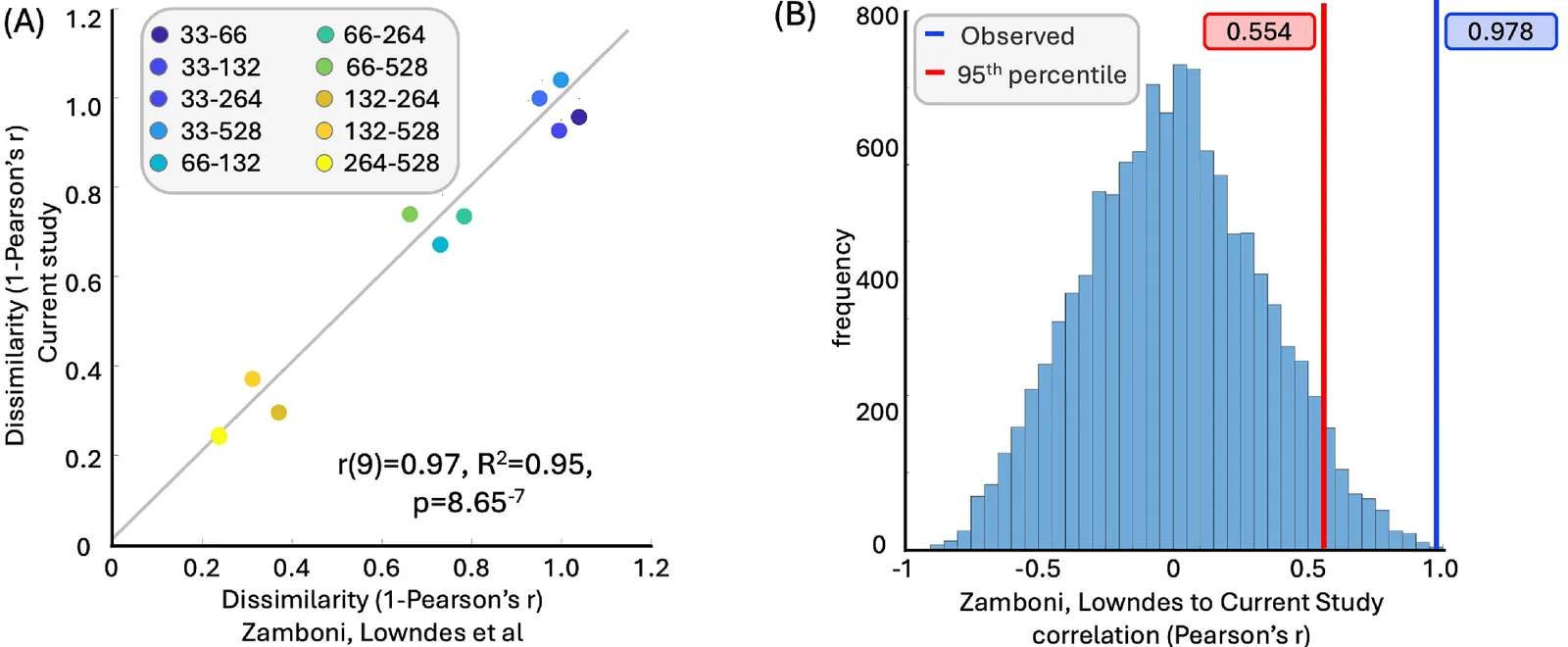
Consistency in time-dependent RDMs across experiments. (A) Scatter plot depicts the linear relationship in the pattern of dissimilarity between time-dependent conditions across both experiments. The pattern of dissimilarity between time-dependent conditions was highly similar (*r*(9) = 0.97) and explained ∼95% of the variance. Condition-pairs are color-coded and can be seen to form three spatially separate clusters. (B) Distribution represents the correlation (Pearson's *r*) between the condition-condition RDM from (Zamboni et al., 2026) and 10,000 randomly shuffled condition-condition RDMs from the current study. The blue line represents the observed correlation (*r* = 0.97). The red lines represent the 95th percentile of the permuted null distribution (*r* = 0.55).

Despite the high similarity in this pattern between experiments, the observed correlation should be interpreted with caution given the low number of data points (*n* = 10). To increase our confidence in this finding we implemented a permutation analysis in which we randomly shuffled the current studies condition-condition RDM before computing the correlation (Pearson's *r*) with the condition-condition RDM from Experiment 1 (Zamboni et al., 2026). This procedure was repeated 10,000 times producing a null distribution of the expected correlations by chance (Figure 6B). We then compared the observed correlation between experiments (*r* = 0.97) against the 95th percentile of the permuted distribution (*r* = 0.55). The observed correlation falls well to the right of the 95th percentile and is therefore highly unlikely to have occurred by chance. This indicates that the pattern of dissimilarity in navigational affordances between the time-dependent conditions is highly repeatable despite substantial changes in scene content.

Pattern of navigational affordance representations generalize from indoor to outdoor scenes: Despite the striking similarity between experiments and the robustness of the correlation, it remains possible that this pattern is driven by some trivial similarity between the two sets of stimuli. As mentioned above, a justifiable critique of much prior work on navigational affordances (Bonner & Epstein, 2017; Dwivedi et al., 2020), including the current group's prior work (Zamboni et al., 2026) has been the use of a relatively small and homogenous set of stimuli that depicted urban indoor environments with clear routes of egress. The current studies stimulus set, while being more heterogeneous than prior work, nevertheless consisted of 50% indoor urban environments. Could the similarity observed between experiments be driven by the overlap in scene content?

To test this possibility, new time-dependent RDMs were derived based on the pairwise dissimilarity in the path trajectories between all outdoor scenes used in the current study (*N* outdoor scenes = 25) for each condition separately (including the baseline). First, we tested whether a significant correlation with the baseline was detectable at 66 ms of stimulus exposure, as was found for the full stimulus set in Experiment 2. Consistent with the full stimulus set, the observed correlation between the baseline and 66ms RDMs of outdoor only scenes (*r* = 0.32) fell well to the right of the permuted null distribution (*r* = 0.09). Next, we computed the pairwise dissimilarity between all outdoor scene time-dependent RDMs. Once again, we observed a striking visual similarity between the pattern of dissimilarity across time-dependent conditions of outdoor scenes (Figure 7A) and the pattern of dissimilarity between time-dependent conditions of indoor urban environments from Experiment 1 (Figure 7A). The same three patterns are also apparent with the 33 ms condition being maximally different from all others, the 66 ms condition occupying a middle dissimilarity and then low dissimilarity between the 132, 264, and 528 ms conditions, respectively. Indeed, the correlation (Pearson's *r*) in the pattern of dissimilarity between time-dependent conditions across both experiments remained very high (*r*(9) = 0.94, *R*^2^ = 0.89, *p* = 3.07^−5^) explaining ∼89% of the variance (Figure 7B). As above, we performed the same permutation analysis by shuffling the outdoor scene condition-condition RDM before correlating the correlation with the condition-condition RDM from Experiment 1 (Zamboni et al., 2026) 10,000k. As shown in Figure 7C, the observed correlation (*r* = 0.94) again fell well to the right of the 95th percentile of the permuted distribution (*r* = 0.55) and is thus unlikely to have been due to chance.

**Figure 7.**
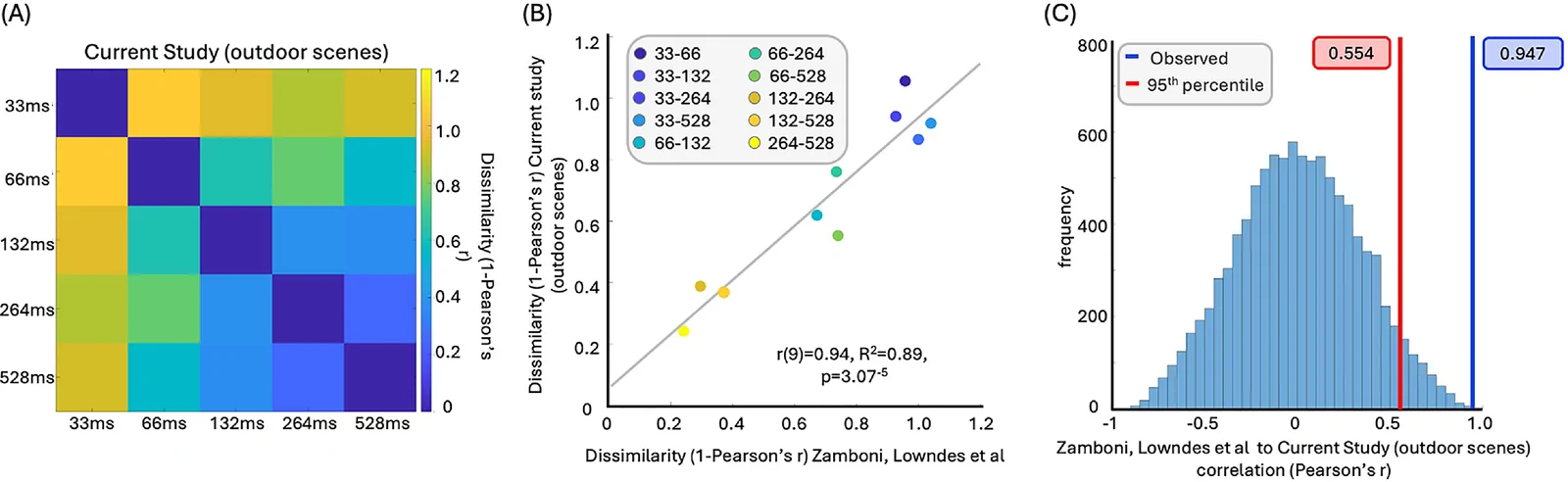
Consistency in time-dependent RDMs across scene content. (A) Time-dependent RDM-RDM matrix of outdoor scenes from the current study. Cells represent the dissimilarity (Pearson's *r*) between all pairs of time-dependent navigational affordance RDMs derived from path trajectories of outdoor environments only. (B) Scatter plot depicts the linear relationship in the pattern of dissimilarity between time-dependent conditions across both experiments. The pattern of dissimilarity between time-dependent conditions was highly similar (*r*(9) = 0.94) and explained ∼89% of the variance. Condition-pairs are color-coded and can be seen to form three spatially separate clusters. (C) Distribution represents the correlation (Pearson's *r*) between the condition-condition RDM from (Zamboni et al., 2026) and 10,000 randomly shuffled outdoor only condition-condition RDMs from the current study. The blue line represents the observed correlation (*r* = 0.94). The red lines represent the 95th percentile of the permuted null distribution (*r* = 0.55).

## Discussion

Prior behavioral work demonstrated that identifying efficient routes of egress can be accomplished with as little as 33-66 ms of stimulus presentation, but that these navigational affordance representations only stabilized with stimulus exposures of 132ms and beyond (Zamboni et al., 2026). Here, we replicate these findings using a more heterogeneous stimulus set that included both indoor urban and outdoor environments (Bartnik et al., 2025).

The ability of the human visual system to extract, process and interpret visual features to inform scene understanding and navigation has been intensely studied for decades (Greene & Oliva, 2009; Groen, Silson, & Baker, 2017; Malcolm, Groen, & Baker, 2016; Potter, 1975; Thorpe et al., 1996). Prior work demonstrated the ability of the human visual system to extract behaviorally relevant visual information at a glance (Greene & Oliva, 2009; Potter, 1975; Thorpe et al., 1996; Zamboni et al., 2026). Here, across both experiments, we observed significant navigational affordance behavior emerging with 33ms of stimulus exposure in Experiment 1 (Zamboni et al., 2026) and 66 ms in Experiment 2. These behavioral results are largely consistent with prior work (Greene & Oliva, 2009) in which stimulus durations of ∼34 ms were sufficient for human observers to discern the navigability of scenes very accurately (75% correct). This consistency is interesting when one considers the very different way in which affordance behavior is operationalized between the current experiments and prior work. In the current work, participants in both experiments were required to identify efficient routes of egress through scenes by tracing these paths with the computer mouse, which is a qualitatively different behavior than a binary decision of whether a scene is navigable or not. Although we were able to observe a significant correlation with as little as 33–66 ms of stimulus exposure, these representations were not strong and only became stable with stimulus durations of 132 ms and beyond. The longer presentation times needed to establish stable representations of navigational affordances, relative to a navigable or non-navigable judgment, likely explain the slightly longer durations reported here. It is also important to acknowledge that these results will be somewhat dependent on the spatial and temporal factors of the current experimental setup and should be interpreted as approximations. Some imprecisions in these measurements are inevitable across labs.

What do we mean by a stable representation? Across both experiments we observed a substantial increase in the baseline correlation between time-dependent conditions at the 66-132 ms comparison compared to all other successive time-dependent pairings (66-132 ms Delta r Exp1 = 0.33; Delta *r* Exp2 = 0.39). Moreover the increase in baseline correlation largely reached an asymptote post 132 ms in both experiments (132-528 ms Delta *r* Exp1 = 0.01; Delta *r* Exp2 = 0.07), indicating a limited benefit to behavior despite quadruple the stimulus exposure. These data suggest that to be maximally beneficial to navigational behavior the human visual system requires as little as 132 ms of stimulus exposure. Such rapid perceptual-behavioral translations likely underpin our ability to navigate seamlessly through environments that are often visually chaotic and highly dynamic (e.g., a busy train station).

A considerable amount of the prior work on navigational affordances has either made use of simplistic artificial computer generated environments (Bonner & Epstein, 2017; Harel et al., 2022; Kamps et al., 2025) or the same set of 50 scenes as was used originally by Bonner and Epstein (Bonner & Epstein, 2017; Dwivedi et al., 2020; Zamboni et al., 2026). This particular set of scenes depicts indoor urban environments with easily identifiable, albeit variable routes of egress. Although the use of these stimuli has been valuable to our understanding of navigational affordance computation and automaticity, it nevertheless limits the generalizability of those findings to this particular sub-category of scenes.

We know from prior work that the visual statistics of indoor and outdoor scenes differ (Groen, Ghebreab, Prins, Lamme, & Scholte, 2013; Groen et al., 2017; Malcolm et al., 2016), as do the neural representations of scenes based on dimensions such as content (person-made/natural), expanse (open/closed) and 3D-structure (Kravitz, Peng, & Baker, 2011; Lescroart & Gallant, 2019; Scrivener, Zamboni, Morland, & Silson, 2024). It was therefore possible that both our prior identification of the very early emergence of navigational affordances (i.e., 3366 ms) and their stability (132 ms of stimulus exposure) could simply reflect some trivial aspect of the visual features within the 50 urban indoor environments used. This possibility is even more likely when one considers that prior computational work on these same 50 scenes showed that ∼80% of the variance in fMRI responses within the OPA could be explained by the visual features in the lower visual field (Bonner & Epstein, 2018), of which OPA overly represents (Scrivener et al., 2024; Silson, Chan, Reynolds, Kravitz, & Baker, 2015; Silson, Groen, Kravitz, & Baker, 2016). As such these stimuli (and the lower half in particular) could have contained very similar visual features because of the materials commonly used to floor indoor urban environments (e.g., plain carpets, wooden floorboards, vinyl flooring). For many people, navigating through daily life likely extends beyond indoor urban environments to include navigating outdoors in both person-made (e.g., a park) and natural (e.g., a wood) settings. It was therefore essential that the same experiment be performed but using a more heterogeneous stimulus set that included both indoor urban and more crucially outdoor environments (Bartnik et al., 2025). The 50 images used in Experiment 2 were selected from a set of 90 to match the previous design. We selected 25 indoor urban environments and 25 outdoor environments. Within the outdoor category, 14 were outdoor person-made scenes (e.g., intersection of a road) and nine were outdoor natural scenes (Bartnik et al., 2025). Importantly, the scenes in Experiment 2 were shown to be more dissimilar to each other in terms of image statistics than the prior set of indoor urban environments.

The consistency in the pattern of results across experiments suggests that representations of navigational affordances emerge rapidly (∼66 ms) and become stable with ∼132 ms of stimulus exposure and that this early emergence and later stability is largely invariant to scene content. It was of course a possibility that this overall consistency was due to the inclusion of indoor urban environments in our Experiment 2 stimulus set, which may have shared visual features with the 50 indoor urban environments of Experiment 1. To rule out this possibility, we performed the same analysis but included the outdoor scenes only. We observed a largely equivalent pattern of results from the outdoor only scenes, which allows us to reject that alternative explanation. Instead, it appears far more likely that 132 ms of stimulus exposure is long enough for the human visual system to form stable representations of navigational affordances that are invariant to scene content. The task used here inherently relies not only the accurate perception of navigational affordances but also an aspect of working memory to reconstruct the paths after mask offset. It is possible that the relatively poor correlations with the baseline observed at 33 and 66 ms stimulus exposure reflect a limited capacity for visual information to be transferred and maintained in working memory, whereas the stability of the effects at 132 ms may reflect a general suprathreshold exposure time. Future work could attempt to tease apart how specific the effects reported here are to the perception of navigational affordances versus more general working memory principles.

Of course, we cannot say with any certainty that 132 ms is the shortest stimulus exposure required to form a stable representation as our experimental design doubled the stimulus exposure at each successive stage (33, 66, 132, 264, and 528 ms). It remains possible that similarly stable representations of navigational affordances could emerge anywhere between 66 and 132 ms of stimulus exposure (a 66 ms window). Future work could systematically vary the timing of stimulus exposure between these two epochs to derive a more precise minimum exposure time. Likewise future work could make use of increasingly large and diverse stimulus sets to more completely test the generalizability of these findings. On the basis of the current work, a strong prediction would be made for a highly consistent pattern regardless of the specific scene content. Much prior recent work on navigational affordances has made use of real-world static 2-D images of scenes (Bonner & Epstein, 2017; Dwivedi et al., 2020; Zamboni et al., 2026), and yet our everyday experiences are not static but highly dynamic. A goal for future work is to examine whether the temporal emergence of navigational affordances in dynamic scenes mirror those reported here. Relatedly, it is important to note the current analysis captures only the overall spatial topography of the path trajectories and does not capture the temporal order in which those paths were drawn. One could imagine, for example, that two individuals shown the same scene may select paths in a different temporal order (e.g., one goes left then right, the other right then left). Under the current framework these would be treated as similar navigational affordance representations, but future work should explore how these affordance representations evolve over time. The current experiment was a between-participant design, selected to be consistent with prior work (Zamboni et al., 2026), but such a choice may not be optimal. Future work could consider applying a within-participant design. This would allow one to compute a measure of within-subject consistency on a scene-by-scene basis directly rather than resorting to correlations of correlations across participants.

Finally, the consistency of findings across both urban indoor and outdoor environments could be interpreted as reflecting a fundamental feature of visual perception. Throughout human history, efficient navigation through the local environment would have been and continues to be an essential part of life. It is therefore perhaps unsurprising that we observe largely identical patterns of behavior across changes in scene content. Taken at face-value, our data suggests that the human visual system can construct navigational affordances rapidly (∼33-66 ms of exposure) and requires as little as 132 ms of stimulus exposure for those representations to stabilize regardless of scene content. Such rapid perception to action planning likely reflects an evolutionary feature of how the visual system extracts visual information to aid scene understanding and navigation specifically.

## References

[bib1] Bartnik, C. G., Sartzetaki, C., Sanchez, A. P., Molenkamp, E., Bommer, S., Vukšić, N., … Groen, I. I. A. (2025). Representation of locomotive action affordances in human behavior, brains, and deep neural networks. *Proceedings of the National Academy of Sciences,* 122(24), e2414005122, 10.1073/pnas.2414005122. PMC12184334 40504155

[bib2] Bonner, M. F., & Epstein, R. A. (2017). Coding of navigational affordances in the human visual system. *Proceedings of the National Academy of Sciences,* 114(18), 4793–4798, 10.1073/pnas.1618228114. PMC5422815 28416669

[bib3] Bonner, M. F., & Epstein, R. A. (2018). Computational mechanisms underlying cortical responses to the affordance properties of visual scenes. *PLoS Computational Biology,* 14(4), e1006111, 10.1371/journal.pcbi.1006111. PMC5933806 29684011

[bib4] Dwivedi, K., Cichy, R. M., & Roig, G. (2020). Unraveling representations in sceneselective brain regions using scene-parsing deep neural networks. *Journal of Cognitive Neuroscience,* 33(10), 2032–2043, 10.1162/jocn_a_01624. PMC7612022 32897121

[bib5] Greene, M. R., & Oliva, A. (2009). The briefest of glances: The time course of natural scene understanding. *Psychological Science,* 20(4), 464–472, 10.1111/j.1467-9280.2009.02316.x. PMC2742770 19399976

[bib6] Groen, I. I. A., Ghebreab, S., Prins, H., Lamme, V.A.F., & Scholte, H. S. (2013). From image statistics to scene gist: Evoked neural activity reveals transition from low-level natural image structure to scene category. *The Journal of Neuroscience: The Official Journal of the Society for Neuroscience,* 33(48), 18814–18824, 10.1523/JNEUROSCI.3128-13.2013. PMC6618700 24285888

[bib7] Groen, I. I., Silson, E. H., & Baker, C. I. (2017). Contributions of low-and high-level properties to neural processing of visual scenes in the human brain. *Philosophical Transactions of the Royal Society B: Biological Sciences,* 372(1714), 20160102. 10.1098/rstb.2016.0102 PMC5206270 28044013

[bib8] Harel, A., Nador, J. D., Bonner, M. F., & Epstein, R. A. (2022). Early electrophysiological markers of navigational affordances in scenes. *Journal of Cognitive Neuroscience,* 34(3), 397–410, 10.1162/jocn_a_01810. 35015877

[bib9] Kamps, F. S., Chen, E. M., Kanwisher, N., & Saxe, R. (2025). Representation of navigational affordances and ego-motion in the occipital place area. *Imaging Neuroscience,* 3, imag_a_00424, 10.1162/imag_a_00424. PMC12319941 40800867

[bib10] Kravitz, D. J., Peng, C. S., & Baker, C. I. (2011). Real-world scene representations in high-level visual cortex: It's the spaces more than the places. *The Journal of Neuroscience,* 31(20), 7322–7333, 10.1523/JNEUROSCI.4588-10.2011. PMC3115537 21593316

[bib11] Lescroart, M. D., & Gallant, J. L. (2019). Human scene-selective areas represent 3D configurations of surfaces. *Neuron,* 101(1), 1, 10.1016/j.neuron.2018.11.004. 30497771

[bib12] Malcolm, G. L., Groen, I. I., & Baker, C. I. (2016). Making sense of real-world scenes. *Trends in Cognitive Sciences,* 20(11), 843–856. 10.1016/j.tics.2016.09.003 PMC5125545 27769727

[bib13] Oliva, A., & Torralba, A. (2006). Chapter 2 Building the gist of a scene: The role of global image features in recognition. In *Progress in Brain Research* (Vol. 155, pp. 23–36). St. Louis: Elsevier. 10.1016/S0079-6123(06)55002-2. 17027377

[bib14] Peirce, J. W. (2007). PsychoPy—Psychophysics software in Python. *Journal of Neuroscience Methods,* 162(1–2), 8–13, 10.1016/j.jneumeth.2006.11.017. PMC2018741 17254636

[bib15] Potter, M. C. (1975). Meaning in Visual Search. *Science,* 187(4180), 965–966, 10.1126/science.1145183. 1145183

[bib16] Scrivener, C. L., Zamboni, E., Morland, A. B., & Silson, E. H. (2024). Retinotopy drives the variation in scene responses across visual field map divisions of the occipital place area. *Journal of Vision,* 24(8), 10, 10.1167/jov.24.8.10. PMC11343012 39167394

[bib17] Silson, E. H., Chan, A. W.-Y., Reynolds, R. C., Kravitz, D. J., & Baker, C. I. (2015). A retinotopic basis for the division of high-level scene processing between lateral and ventral human occipitotemporal cortex. *Journal of Neuroscience,* 35(34), 34, 10.1523/JNEUROSCI.0137-15.2015. PMC4549403 26311774

[bib18] Silson, E. H., Groen, I. I. A., Kravitz, D. J., & Baker, C. I. (2016). Evaluating the correspondence between face-, scene-, and object-selectivity and retinotopic organization within lateral occipitotemporal cortex. *Journal of Vision,* 16(6), 6, 10.1167/16.6.14. PMC4898275 27105060

[bib19] Thorpe, S., Fize, D., & Marlot, C. (1996). Speed of processing in the human visual system. *Nature,* 381(6582), 520–522, 10.1038/381520a0. 8632824

[bib20] Zamboni, E., Lowndes, R., Aveyard, R., Scrivener, C. L., Teed, J. A., Ma, Y., … Silson, E. H. (2026). Early spatiotemporal dynamics of navigational affordance coding in the dorsal visual cortex. *Nature Communications,* 17(1), 1361, 10.1038/s41467-025-68111-7. PMC12876848 41507190

